# Effects of Exogenous Neuroglobin (Ngb) on retinal inflammatory chemokines and microglia in a rat model of transient hypoxia

**DOI:** 10.1038/s41598-019-55315-3

**Published:** 2019-12-11

**Authors:** Sai Bo Bo Tun, Veluchamy Amutha Barathi, Chi D. Luu, Myoe Naing Lynn, Anita S. Y. Chan

**Affiliations:** 1 Singapore Eye Research Institute, Singapore National Eye Centre, The Academia, 20 College Road, Discovery Tower Level 6, Singapore, Singapore 169856; 2 Department of Ophthalmology, Yong Loo Lin School of Medicine, National University of Singapore, 1E Kent Ridge Road Level 11, NUHS Tower Block, Singapore, Singapore 119228; 3 Ophthalmology Academic Clinical Research Program, DUKE-NUS Graduate Medical School, 8 College Road, Singapore, Singapore 169857; 4 Centre for Eye Research Australia, Royal Victorian Eye and Ear Hospital, Melbourne, Victoria Australia; 5 Department of Surgery (Ophthalmology), The University of Melbourne, Melbourne, Victoria Australia; 6 Singapore National Eye Centre, 11 Third Hospital Ave, Singapore, Singapore 168751; 7 Singapore General Hospital, Department of Anatomical Pathology, The Academia, 20 College Road, Diagnostic Tower Level 10, Singapore, Singapore 169856

**Keywords:** Cell death and immune response, Neuroscience, Neurophysiology

## Abstract

Neuroglobin is an endogenous neuroprotective protein. We determined the safety of direct delivery of Neuroglobin in the rat retina and its effects on retinal inflammatory chemokines and microglial during transient hypoxia. Exogenous Neuroglobin protein was delivered to one eye and a sham injection to the contralateral eye of six rats intravitreally. Fundus photography, Optical Coherence Topography, electroretinogram, histology and Neuroglobin, chemokines level were determined on days 7 and 30. Another 12 rats were subjected to transient hypoxia to assess the effect of Neuroglobin in hypoxia exposed retina by immunohistochemistry, retinal Neuroglobin concentration and inflammatory chemokines. Intravitreal injection of Neuroglobin did not incite morphology or functional changes in the retina. Retinal Neuroglobin protein was reduced by 30% at day 7 post hypoxia. It was restored to normoxic control levels with intravitreal exogenous Neuroglobin injections and sustained up to 30 days. IL-6, TNFα, IL-1B, RANTES, MCP-1 and VEGF were significantly decreased in Neuroglobin treated hypoxic retinae compared to non-treated hypoxic controls. This was associated with decreased microglial activation in the retina. Our findings provide proof of concept suggesting intravitreal Neuroglobin injection is non-toxic to the retina and can achieve the functional level to abrogate microglial and inflammatory chemokines responses during transient hypoxia.

## Introduction

Neuroglobin (Ngb) is an endogenous heme protein found abundantly in the retina^1–4^. Previously, we demonstrated its neuroprotective role during transient high intraocular pressure (IOP) induced retinal ischemia in transgenic neuroglobin overexpressing mice retina through the abrogation of mitochondrial oxidative stress^4^. Currently, cell penetrating peptide (CPP)-Ngb coupled peptides and chimeric Zebrafish-Human Ngb protein were the reported delivery modalities shown to increase Ngb expression *in-vivo* in order to study its role in neuroprotection^5^. However, the small size of Ngb (17 kDa) suggests that direct intra-retinal penetration may be possible^6,7^. This pilot study aimed to demonstrate that a simple exogenous Ngb protein delivery via intravitreal (IVT) injections is safe and able to elevate retinal Ngb protein levels. Our secondary aim was to determine the retinal effects of exogenous IVT Ngb on inflammatory cytokines and microglial activation in rats exposed to transient hypoxia in order to establish proof of concept that direct Ngb protein injections may be a potential delivery modality for the study of its neuroprotective effects.

## Results

### Exogenous Ngb is safe to be delivered intravitreally in rats

**Figure 1:**
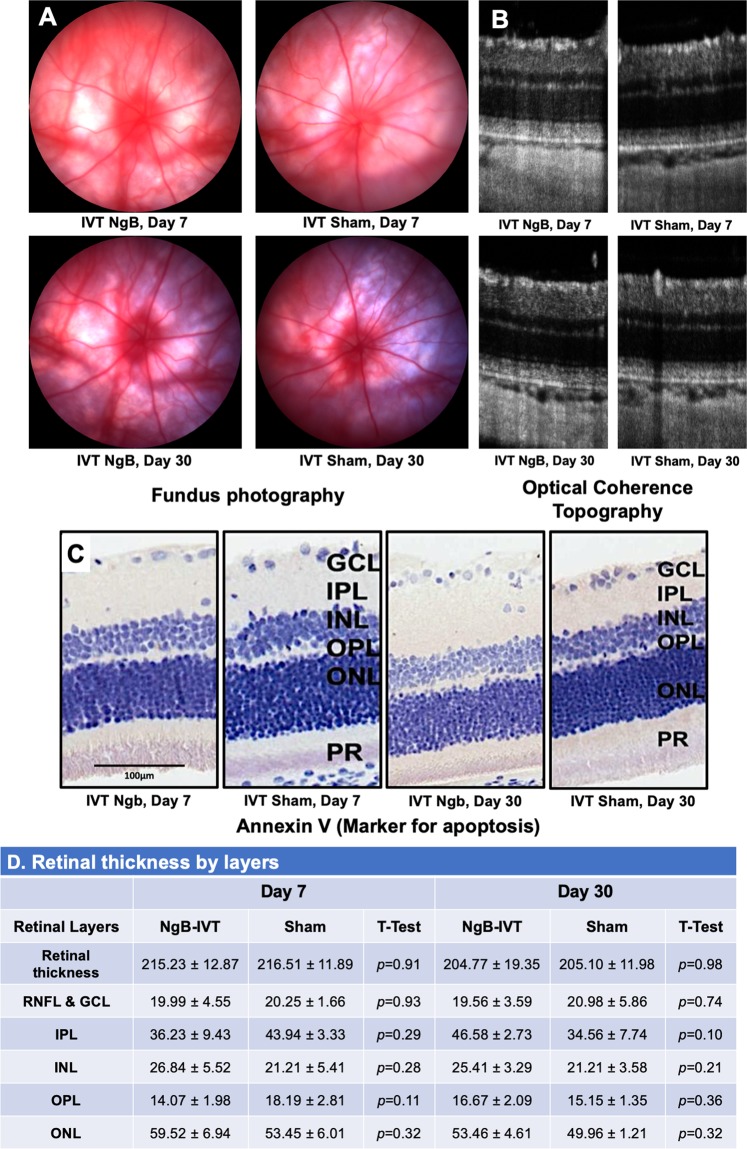
Fundus and retinal thickness comparison between non-Ngb treated and Ngb treated retinae. (**A**) Fundus images after the Intravitreal injection of Ngb showing no evidence of inflammation, vasculitis or endophthalmitis. (**B**) Optical Coherence Topography after Intravitreal injection of Ngb showing no significant retinal thinning. (**C**) Annexin V immunohistochemistry using diaminobenzidine showing no increased apoptosis post intravitreal Ngb injection (performed in the presence of satisfactory positive controls, data not shown). (**D**) Morphometric analysis of individual retinal layers showing no statistical significance between the Ngb injected retinae and that of the sham controls. (RNFL: Retinal Nerve Fiber Layer, GCL: Ganglion Cell Layer, IPL: Inner Plexiform Layer, INL: Inner Nuclear Layer, OPL: Outer Plexiform Layer, ONL: Outer Nuclear Layer, PR: Photoreceptor Layer). Statistical Analysis: Simple T-Test, *p* < 0.05 taken as statistical significance. The values are in means ± SD.

**Figure 2:**
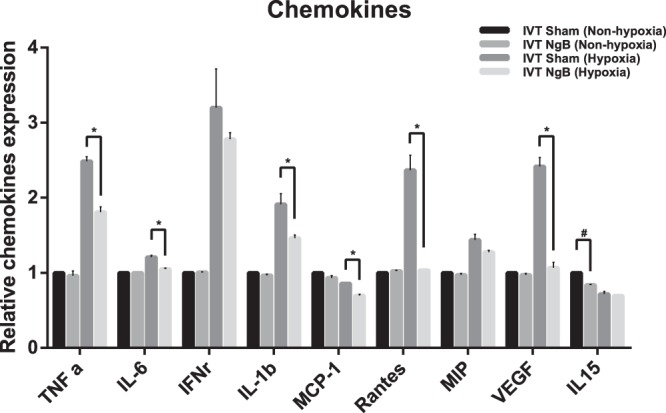
Mean retinal concentration of inflammatory and angiogenic chemokines. Majority of the chemokines were increased at day 30 after exposure to transient hypoxia. There was a significant decreased in TNFα: Tumour necrosis factor alpha, IL-6: Interleukin 6, IL-1B: Interleukin 1 Beta, MCP-1: Monocyte chemoattractant protein-1, RANTES: Regulated on Activation Normal T Cell Expressed and Secreted, VEGF: Vascular Endothelial Growth Factor in the Ngb treated retinae in comparison with the non-treated control retinae of post hypoxic attack. The other inflammatory chemokines, INFγ: Interferon Gamma, MIP: Macrophage Inflammatory Protein, IL-15: Interleukin 15 were also lowered in the Ngb treated retinae. Statistical Analysis: Simple T-Test, *,# *p* < 0.05 (error bars: standard deviation of the mean).

On days 7 and 30 post injection, fundus images revealed no evidence of vasculitis, vitritis, retinitis and retinopathy in the injected eyes (Fig. 1A). Functional studies using electroretinogram (ERG) also showed no significant difference in a-wave (Day 7, Ngb: 149.63 + 56.84, BSS: 159.84 + 30.81, *p* = 0.72) (Day 30, Ngb: 168.75 + 54.31, BSS: 142.4 + 27.92, *p* = 0.33) and b-wave amplitudes (Day 7, Ngb: 488.65 + 171.86, BSS: 570.2 + 150.6, *p* = 0.42) (Day 30, Ngb: 563.02 + 140.12, BSS: 492.98 + 66.04, *p* = 0.31) of the dark-adapted maximal response (10 cd.s.m^−2^). Histology displayed no apparent change in each individual layer thickness of the retina (*p* > 0.05) between sham controls and Ngb treated eyes (Fig. 1B,D). Apoptosis detection using annexin V, an apoptotic marker (in the presence of a positive control), was not seen in the retinae of both groups (Fig. 1C). There was a slight reduction of most of the chemokines expression in Ngb injected retinae compared to the controls. (Fig. 2).

### IVT Ngb protein can restore the depleted Ngb in transient hypoxia exposed retinae

**Figure 3:**
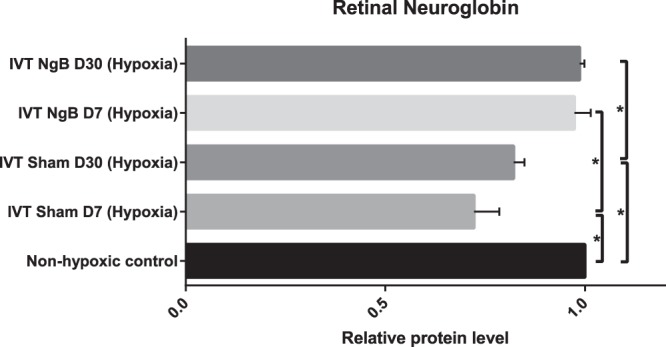
Mean retinal Ngb Concentration. Intravitreal Ngb treated hypoxic retinae showed statistically significant increased levels of mean retinal Ngb concentration at day 7, and this was sustained till day 30 post hypoxia. There was a significant reduction of mean Ngb retinal concentration after the hypoxic attack at both day 7 and day 30. Statistical Analysis: Simple T-Test, *p < 0.05 (error bars: standard deviation of the mean).

To determine if exogenous Ngb protein via IVT treatment could replace depleted endogenous levels during hypoxic insult, we subjected 12 rats to transient hypoxia, and the left eyes received BSS sham injections while the contralateral eyes received Ngb injections. After exposure to hypoxia, retinal Ngb concentration was reduced significantly (*p* < 0.05) by 27.8% at day 7 and 17.9% at day 30 respectively compared to non-hypoxic retinae (Fig. 3). After two doses of IVT Ngb injections at day 0 (pre-treatment before hypoxia exposure) and day 3 (post hypoxia exposure), retinal Ngb level was increased by 35% (*p* < 0.05) at day 7 and 20% (*p* < 0.05) at day 30 post hypoxia compared to sham control eyes. These levels were similar to that of the non-hypoxic retinal Ngb level. (Fig. 3).

### IVT Ngb attenuates retinal chemokines and microglia leading to reduction of apoptosis in the post transient hypoxic retinae

**Figure 4:**
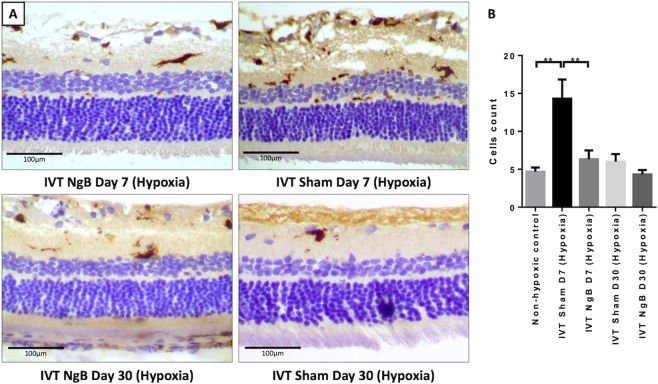
Microglia (IBA1) immunohistochemistry staining in the retina exposed to hypoxia. (**A**) Immunohistochemistry staining with anti-IBA1 antibody specific for microglial/macrophage. (**B**) There was a significant downregulation of microglial in the NgB treated hypoxic retina at day 7, which further reduced at day 30 compared to control retinae exposed to transient hypoxia. Statistical Analysis: Simple T-Test, **p < 0.01 (error bars: standard deviation of the mean).

**Figure 5:**
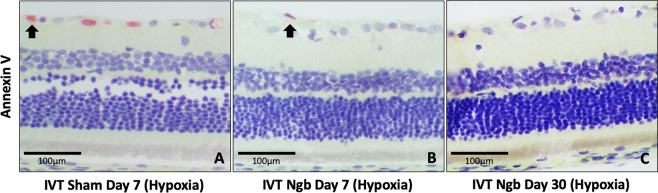
Annexin immunohistochemistry staining (Red chromogen). (**A**) Hypoxic retina at Day 7 showed some apoptosis of the retinal ganglion cells (Black arrow). (**B**) Ngb treated hypoxic retina at Day 7 showed reduction of apoptosis, with rare retinal ganglion cells with positive staining (Black arrow) (**C**) Ngb treated hypoxic retina at Day 30 showed no apoptosis.

In a comparison of the retinal inflammatory chemokines, we found a significant reduction (*p* < 0.05) of TNFα (Tumour Necrosis Factor alpha), IL-6 (Interleukin 6), IL-1B (Interleukin 1 Beta), RANTES (Regulated on Activation Normal T Cell Expressed and Secreted), MCP-1 (Monocyte Chemotactic Protein 1) and VEGF (Vascular Endothelial Growth Factor) in IVT Ngb treated retinae, at 30 days post exposure to transient hypoxia compared to the non-treated hypoxic control (Fig. 2). INFγ: Interferon Gamma, MIP: Macrophage Inflammatory Protein, IL-15: Interleukin 15 were also lowered in the Ngb treated retinae. Histology analysis using an activated microglial marker, Ionized calcium binding adaptor molecule 1 (IBA1) showed microglial were activated and significantly (*p* < 0.05) increased in hypoxia induced sham treated retinae at day 7, which was lowered almost to normal level by Ngb treatment (Fig. 4). Apoptosis staining using Annexin V revealed that the retinal ganglion cells apoptosis is reduced in the Ngb treated hypoxic retinae compared to the untreated hypoxia exposed retinae at day 7 (Fig. 5).

## Materials and Methods

### Intravitreal injection of exogenous Ngb protein

A total of 18 Wistar rats aged between 50–60 days were used for this experiment. All experimental procedures and animal handlings were performed according to the guidelines and approval from the SingHealth Institutional Animal Care and Use Committee (IACUC). In order to determine whether exogenous Ngb could be delivered without detrimental effects to the eye, six rats received double injections of 5 ul (1 mg/ml) of Neuroglobin protein (ProSpec-Tany Technogene Ltd, CYT-450) intravitreally in the right eyes and balanced salt saline (BSS) in the left eyes at day 0 and day 3 using previously published methods of IVT delivery^8^. Intravitreal injections were performed under sterile conditions using a 31 G Hamilton needle mounted on a 10 ul glass syringe (Hamilton Co., Reno, NV).

### Assessment of ocular health after exogenous Ngb intravitreal injection

On days 7 and 30, fundus photography and OCT images were taken using micron IV fundus camera (Phoenix Research Laboratories, USA) for both Ngb and sham treated eyes. Clinical signs of vitritis, retinitis and optic neuropathy were examined by an experienced clinician. OCT images were analyzed using InSight software V. 1.1 to determine retinal thickness and sign of retinal degeneration. Electroretinogram (ERG) was recorded after overnight dark-adaptation (>12 hours) using corneal monopolar electrodes and an Espion system (Espion, Diagnosys LLC, USA). The stimulus intensity ranging from −3.0 to 1.0 log cd.s.m^−2^ was used. The harvested retinae were used to analyze the expression of the chemokines, and histology. The eyes were fixed in 10% neutral buffered formalin immediately after enucleation and embedded in paraffin blocks for histology. These were sectioned into four-micron sections and stained with Hematoxylin and Eosin staining (H&E) for morphology, retinal thickness and features of inflammation (presence of macrophages and lymphocytes). Annexin V immunohistochemistry (abcam, ab14196, 1:200 dilution), a marker for apoptosis, was performed to detect apoptotic cells.

### Transient hypoxia exposure and inflammatory chemokines assay

To determine the effects of intravitreal Ngb injection on post hypoxia pro-inflammatory and chemotactic chemokines (TNFα, MCP-1, IL-6, IL-1B, RANTES, INFγ, IL-15, and VEGF), 12 rats received the intravitreal injection of 5 ul of Neuroglobin protein (1 mg/ml, ProSpec-Tany Technogene Ltd, CYT-450) in the right eyes and BSS in the left eyes on day 0 (pre-treatment before hypoxia exposure) and day 3 (post hypoxia exposure). On day 1, these 12 rats were subjected to hypoxia using a previously published method of hypoxia^9,10^. In brief, the rats were placed in a decompression (hypobaric) chamber (Galaxy® 170 R CO2 incubator, New Brunswick) filled with a gas mixture of 7% oxygen and 93% nitrogen for 2 hours and then allowed to recover under normoxic conditions. The retinae from all 18 rats, 36 globes were harvested for analysis of pro-inflammatory cytokines and VEGF (Rat Inflammation and Oxidative Stress ELISA Strip, Signosis, EA-1201 and EA-1501), Ngb quantification by ELISA kit (Rat Neuroglobin ELISA Kit, MyBiosource, MBS704958) and immunohistochemical staining with annexin V (abcam, ab14196, 1:200 dilution), and anti-IBA1 antibodies (abcam, ab178847, 1:200 dilutions) for the detection of activated microglial cells. Activated microglial cells count in the retina was quantified manually from three random sections from each group immunohistochemically stained with anti-IBA1 antibodies.

## Discussion

Intravitreal protein delivery of anti-VEGF has become the mainstay of choroidal neovascularisation in age related macular degeneration and macular edema treatment^11^. This has paved the way for the development of other Intravitreal drugs^6,7^. In the retina, several structures: the outer limiting membrane (OLM), plexiform layers and inner limiting membrane (ILM) are known to limit the free diffusion of the proteins^12^. At these sites, although occludin tight junctions may limit the penetration of intravitreally injected proteins, studies have also shown that in a disease state such as diabetic retinopathy or hypoxia, these tight junctions may be damaged allowing protein penetration^12^. Other studies have also shown that direct intravitreal injection of an exogenous small protein such as recombined nerve growth factor (rNGF, 13.2 kDa) can penetrate the retina to mediate its neuroprotective effects. These findings underlie the rationale of our study aimed to determine the safety and retinal effects of intravitreal exogenous Ngb, a small 17 kDa protein during transient hypoxia.

This pilot study demonstrates proof of concept that direct intravitreal injection of exogenous Ngb protein is safe. There was no significant inflammation from the injection (BSS sham injection) nor as a response to the protein itself. No structural change was seen on fundus examination (Fig. 1), and there was no evidence of retinal dysfunction. As with all intravitreal injections performed under aseptic techniques, the standard risk of endophthalmitis exists, but, in our study, only one eye (sham control injected with BSS) out of 36 eyes developed endophthalmitis.

**Figure 6:**
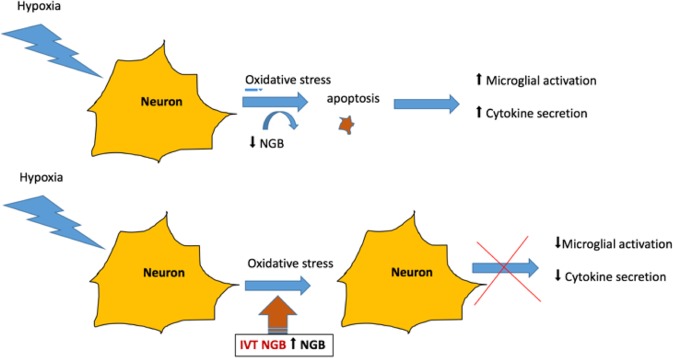
Effect of Ngb as a neuronal protectant (**A**) Transient hypoxia results in a decline of Ngb and in turn leads to oxidative stress-induced apoptosis, which triggers microglial activation and chemokines secretion. (**B**) Transient hypoxia with IVT Ngb injections to increase Ngb levels results in decreased oxidative stress-induced apoptosis and thus reduced microglial activation and cytokine secretion.

Retinal hypoxia underlies numerous ophthalmological conditions such as retinal vascular occlusions, diabetic retinopathy, retinopathy of prematurity and uveitis^10,13–16^. During transient hypoxia, an endogenous neuroprotective mechanism exists in the retina that prevents apoptosis from minor or transient hypoxic insults. These mechanisms include the secretion of inflammatory chemokines such as TNFα, IL-1B, IL-6, IL-8, MCP-1, RANTES and VEGF^10,11,13–16^. Previous studies using this transient hypoxia rodent model have shown that early activation of microglia in response to hypoxia results in the secretion of some of these cytokines such as TNFα. This, in turn, triggers a cascade of chemokines production such as IL-8, VEGF, MCP-1, and IL-1B^9,10^. In this pilot study, intravitreal Ngb treated eyes were found to have decreased microglial activation (Fig. 4) and was accompanied by a significant reduction of TNFα, IL-6, IL-1B, RANTES, MCP-1 and VEGF (Fig. 2), suggesting that intravitreal Ngb injections may abrogate the inflammatory cell response during transient hypoxia. Neuroglobin has been reported to be expressed in cultured astrocytes, the predominant glial cells in the brain. Increased Ngb protein, in a sense oligonucleotide transfected astrocytes resulted in decreased apoptosis after five hours of ischemic insult^17^. Neuroglobin is reported to play a regulatory role in neuronal signalling pathways by preserving mitochondrial ATP production and by scavenging ROS. During hypoxia, Ngb overexpressing primary neuron cultures diminish hypoxia-induced polarisation and mitochondrial aggregation in neurons which reduced the mitochondrial permeability transition pore opening and decreased cytochrome C release after hypoxia, resulting in decreased neuronal apoptosis^18^. Previous studies have also shown the neuroprotective effects of Ngb via the abrogation of  mitochondrial oxidative stress, which leads to reduced apoptosis and neuronal cell death^4,19^. Ngb is predominantly expressed in the ganglion cells and photoreceptors of the retina^20^. During hypoxic insult, the exogenous IVT Ngb injections decreased the ganglion cells apoptosis (Fig. 5), which in turn reduced the activation of microglial cells and inflammatory chemokines level (Figs. 2 and 4). This potential mechanism of action is summarised in Fig. 6.

As limited intraocular therapy is currently available for the treatment of hypoxic retinal diseases^6^, this study using direct exogenous IVT injection of Ngb protein supports previous studies findings demonstrating neuroprotective effects of Ngb but without the use of viral vectors, CPP-Ngb coupled proteins or chimeric proteins. In this pilot study, we had limitations that include small sample sizes and limited tissues for further molecular analysis on the mechanism of action. However, our primary aim was to demonstrate the safety and efficacy of IVT Ngb injections. We were also able to demonstrate the potential therapeutic effects against transient hypoxia that would need further evaluation for translation to potential therapy.

## Data Availability

All materials and data are available upon request with no restriction.
